# Visual attention and processing function in relation to executive functioning in very preterm–born children aged 3 years: a prospective cohort study

**DOI:** 10.1007/s00431-024-05720-2

**Published:** 2024-08-17

**Authors:** Alja Bijlsma, Maud M. van Gils, Victoria A. A. Beunders, Irwin K. M. Reiss, Koen F. M. Joosten, Johan J. M. Pel, Marlou J. G. Kooiker, Marijn J. Vermeulen

**Affiliations:** 1 Department of Neonatal and Pediatric Intensive Care, Division of Neonatology, Erasmus MC-Sophia Children’s Hospital, Rotterdam, the Netherlands; 2 Vestibular and Oculomotor Research Group, Department of Neuroscience, Erasmus MC, Rotterdam, the Netherlands; 3 Department of Neonatal and Pediatric Intensive Care, Division of Pediatric Intensive Care, Erasmus MC-Sophia Children’s Hospital, Room Sk-3280, PO Box 2060, 3000 CB Rotterdam, the Netherlands; 4 Royal Dutch Visio, Center of Expertise for Blind and Partially Sighted People, Amsterdam, the Netherlands

**Keywords:** Preterm children, Eye tracking, Visual orienting functions, Visual processing dysfunctions, Executive functions

## Abstract

The ability to orient attention to one’s environment is a prerequisite for developing executive functions (EF) from preschool age. Very preterm children are vulnerable for delays in visual orienting function (VOF) and EF deficits. This study aimed to investigate associations between objective VOF and subjective parent-reported EF in very preterm–born children at 3 years corrected age (CA). In a prospective cohort study (BOND) involving 90 children born < 30 weeks, VOF and EF were assessed using an eye tracking–based method and BRIEF-P questionnaire. Associations between abnormal VOF (viewing reaction times) and EF scores (BRIEF-P scores) were studied using multivariable regression. Using a modified Delphi method, a subset of eight items related to VOF and EF was explored. Abnormal VOF was observed in 31% of the children and abnormal global EF composite scores in 41%. Abnormal VOF was not associated with global or domain-level EF scores. However, children with abnormal VOF more frequently had EF problems related to attentional behavior: “easily sidetracked” (OR 4.18 (CI: 1.21–14.41), *p* = 0.02) and “short attention span” (OR 4.52 (CI: 1.34–15.22), *p* = 0.02). *Conclusion*: Although abnormal VOF was not associated with global, parent-reported, EF at 3 years CA, secondary analyses did show a relation to specific attention and concentration span items. Further research is needed to study the role of VOF in objectifying preschool EF assessments in very preterm born children.

**Table:** 

What is known: • *Very preterm children are vulnerable for executive function (EF) deficits and for delays in visual orienting function, as compared to term-born children* • *The ability to orient attention to one’s visual environment is an important prerequisite for EF early in life and is easy to measure objectively with visual orienting function (VOF) assessment*
What is new: • *VOF measurement at 3 years of age relates to parent-reported attention and concentration span items, important aspects of executive functions* • *Future work should explore the clinical additional value of early VOF measurement in children at risk for EF deficits*

**Supplementary Information:**

The online version contains supplementary material available at 10.1007/s00431-024-05720-2.

## Introduction

Children born very preterm are at risk of impaired brain growth and development [1], which may lead to neurodevelopmental impairments, creating challenges in motor, cognitive, sensory, or behavioral functioning starting in early and late childhood [2, 3]. At older ages, more complex functions such as concentration, inhibition, emotional self-regulation, and planning, collectively called executive functioning (EF), are needed for goal-directed behavior. EF (also called executive control or cognitive control) refers to a family of top-down mental processes needed when you have to concentrate and pay attention, where automatic action or relying on instinct or intuition would be inappropriate, insufficient, or impossible [4]. A growing body of evidence shows that preterm birth is linked to impaired EF [5]. Later in life, EF dysfunctions can threaten normal academic achievements, social participation, and well-being [6]. Early recognition of EF problems is vital for providing timely developmental support, potentially improving long-term health and well-being, and benefiting families, communities, and society [6].

While measures for separate subfunctions of EF are available for preschoolers, they lack age-related norms, hampering objective assessment and clinical interpretations [7]. Therefore, parent-reported questionnaires on EF-related behavior in daily life provide important additional information [8]. Studies on objective measures of EF and the parent-reported BRIEF-P found that complementary and extensive assessments of EF in preterm and full-term preschoolers provide valuable insights into behavior problems and executive function impairments in these groups [9, 10]. A more simple objective EF assessment may be found in disentangling the underlying lower-order cognitive processes necessary for development and proper exhibition of EF, such as speed of information processing and attentional functions [11]. During the first 2 years of life, deficits in visual attention progress from basic reflexive functions to difficulties in more advanced endogenous attention processes, such as focused attention [12]. Visual orienting function (VOF), the ability to orient attention to environmental events, can be assessed at a young age (infancy and early childhood), using eye tracking–based methods [3, 13–17]. Such an eye tracking–based VOF method has been applied to assess visual attention and the first stages of visual information processing. VOF is measured as the timing of a child’s first gaze response to visual information and can be indicative for visual attention and processing quality [13]. Studies in very preterm preschool and school-aged children have not only shown altered attention network development and delays in VOF[3, 13–17], but also revealed that delays in VOF at 1 year corrected age (CA) are associated with lower Bayley-III cognitive and motor scores at 2 years CA. This highlights the importance of visual attention and speed of information processing for broader neurodevelopment after preterm birth [15, 18].

Currently, no simple, objective methods are available to assess executive function in preschool children born very preterm. To address this gap, this study aims to:

1. Describe VOF and EF of children born very preterm at 3 years CA.
2. Investigate the relation between objectively measured VOF results and more subjectively reported EF scores.
3. Explore whether VOF is predictive of specific EF in daily life.

Given the association between preterm birth and deficits in visual attention, we hypothesized that preterm birth is related to abnormal VOF at 3 years, which, in turn, is related to compromised aspects of EF.

## Material and methods

### Participants

This study is part of the BOND project, an ongoing prospective observational birth cohort study. The BOND study included 142 very preterm infants, who were born before 30 weeks gestational age (GA) and admitted within 48 h after birth to the Neonatal Intensive Care Unit (NICU) between 2014 and 2017 [19]. Infants with congenital anomalies, early severe brain injury (intraventricular hemorrhage (IVH) grade > II or post-hemorrhagic ventricular dilatation (PHVD)), congenital infection, or perinatal asphyxia (umbilical cord pH < 7.00 and Apgar score below 5 after 5 min) were excluded. Children with retinopathy of prematurity (ROP) > grade 3, as assessed by a pediatric ophthalmologist, were excluded. All remaining participants were invited for a study visit at 3 years CA between February 2018 and November 2020. The VOF assessment and parental BRIEF-P questionnaire were part of the general study protocol applied to all participants. The Medical Ethics Committee approved this study (MEC-2014–379). Written informed consent was obtained from all parents.

### Patients’ medical reports

Patient data were collected prospectively from the electronic medical records and parent-reported follow-up questionnaires. Ethnicity was classified as “Western-European” or “non-Western.” Level of parental education level (lower, middle or higher) was based on both parents, retrieved from questionnaires [20]. Age- and sex-adjusted standard deviation scores (SDSs) for birth weight were calculated with the Fenton Growth Chart Calculator [21]. Small for gestational age (SGA) was defined as birth weight < 10th percentile [21]. Age and sex-corrected SDS for weight and weight-for-height SDS were calculated using Dutch reference values [22]. To account for persistent bias due to preterm birth, age was corrected for gestational age, in line with national policy and the literature [23].

### VOF assessment

The participants underwent an eye tracking–based assessment at 3 years CA using a method previously described [13, 17, 24]. During the 7-min test, the child was seated on a chair or parent’s lap at 60-cm distance from a 24-in monitor with an integrated infrared eye-tracking system sampling at 60 Hz (Tobii T60XL; Tobii Corporation, Danderyd, Sweden). The system measured the gaze position of each eye separately with a latency of 30 ms (ms). After a calibration procedure, children’s viewing reactions were recorded during the presentation of a preferential looking paradigm on the monitor [25]. Various visual stimuli with distinctive target areas were randomly presented, to assess visual attention orienting and various types of visual processing [14]. Recorded eye movement data were analyzed offline using Matlab-based software (Mathworks Inc., Natick MA, USA), with a focus on reflexive, externally triggered viewing reactions to the different visual stimuli [14, 17, 26]. For each stimulus presentation, it was recorded whether the child detected the stimulus’ target area, and it was analyzed how fast the eyes reached the target (average reaction time to fixation, RTF) [27]. A 5-point Likert scale was used to monitor the level of attention, fatigue, and restlessness/mobility, with option (1) representing “not at all” to option (5) representing “all the time.”

We analyzed viewing reactions to two stimuli that were previously found to trigger abnormal RTFs in preterm children at 1 year, namely, Motion and Form (measures of motion and form processing) [14]. We also analyzed viewing reactions to cartoon and contrast stimuli (measures of general visual attention orienting and contrast processing) (Supplemental Figure S1) [14]. To reach previously reported high reproducibility rates, RTF results were deemed reliable and included in the analyses if the child detected at least 20% of stimulus presentations, i.e., for the cartoon stimulus, a minimum of three presentations had to be seen [27]. For each child, RTFs were classified as either normal (within 95% confidence interval (CI)) or abnormal (outside the CI) based on a previously described normative reference sample of age-matched full-term born and typically developing controls. Their + 2 SD limits at 3 years were 274 ms for cartoon, 433 ms for contrast, 1066 ms for Form, and 898 ms for Motion [24]. Children with all reactions within the norm were categorized as having “normal VOF”; those with a delayed reaction to at least one of the stimuli were categorized as having “abnormal VOF.”

### Executive function (BRIEF-P questionnaire)

The parent(s)/caregiver(s) were asked to complete the Dutch paper version of the BRIEF-P, a commonly used questionnaire for preschool-aged children (2 to 5 years and 11 months) [8]. They rated 63 items on EF in the context of the everyday environment, as “1” = never a problem, “2” = sometimes a problem, or “3” = often a problem in the past 6 months [8]. Scores were created for the five subscales (inhibit, shift, emotional control, working memory, and plan/organize), three broad indexes (inhibitory self-control, flexibility, and emergent metacognition), and one composite score and two validity scores (inconsistency and negativity). To standardize for age and gender, norm-based *T*-scores were calculated (mean 50, standard deviation (SD)) [8], with higher scores indicating more difficulties with EF [28]. A normal score was defined as a *T*-score below 60; a subclinical score between 60 and 65 and *T*-scores at or above 65 may indicate clinically significant difficulties. The BRIEF-P manual provided Dutch Reference Norms, where 6% of the healthy term born age-matched children have a clinical Global Executive Composite score [28].

We emphasize that VOF provides objective measurements of visual attentional processes, while the BRIEF-P captures parent-reported behaviors related to executive function (EF). By integrating these two assessment tools, we aimed to achieve a more comprehensive understanding of (an important part of) EF in very preterm children than would be possible with either measure alone. To explore whether VOF is associated with specific executive tasks, while limiting type I error, we focused on a selection of specific items as secondary analysis. To prioritize clinically relevant items, we used a modified Delphi technique, where the selection was made based on expert opinion without knowledge of the data. In December 2022, six experts from different fields (AB, MJV, KFMJ, JJMP, MMvG, and MJGK) individually evaluated all 63 items and identified the 8–12 items expected to be (most strongly) associated with VOF [29]. Items mentioned by > 66% of the panel members were selected for further analysis. This resulted in a selection of eight items (covered by four subscales), as listed in Supplemental Table S1.

### Statistical analysis

Comparisons of patient characteristics between the groups were done using Mann Whitney *U* or chi-square tests. Follow-up data are reported as medians (interquartile ranges (IQR)) and numbers (percentage of total group) for the neonatal and visual parameters as means and SD for the executive functions.

To study the association of abnormal versus normal VOF (independent variables) and EF (composite score, subscales, and index scores as dependent factors), multiple linear regression analyses were applied, with complete case analysis (one incomplete). First, basic models were run, corrected for the CA at assessment. Then, a confounder model was run, additionally corrected for sex, birth weight, and parental education level. These factors were selected based on literature [11, 14, 20, 30] and a directed acyclic graph (Supplemental Figure S2). Birth weight was considered a proxy for various perinatal and clinical characteristics (such as GA, dysmaturity, sepsis, bronchopulmonary dysplasia, duration of hospital admission). Parental education level was considered a proxy for socio-economic status and lifestyle. The secondary, explorative analyses of the association between BRIEF-P items (items scored by parent(s) as “no/sometimes” or “often”) and VOF measures were based on logistic regression, using a basic model and a confounder model as described above. *P* values (two-tailed) below 0.05 were considered statistically significant. Statistical analysis was performed using SPSS version 25.0 (IBM SPSS Statistics, Chicago, IL).

## Results

**Fig. 1:**
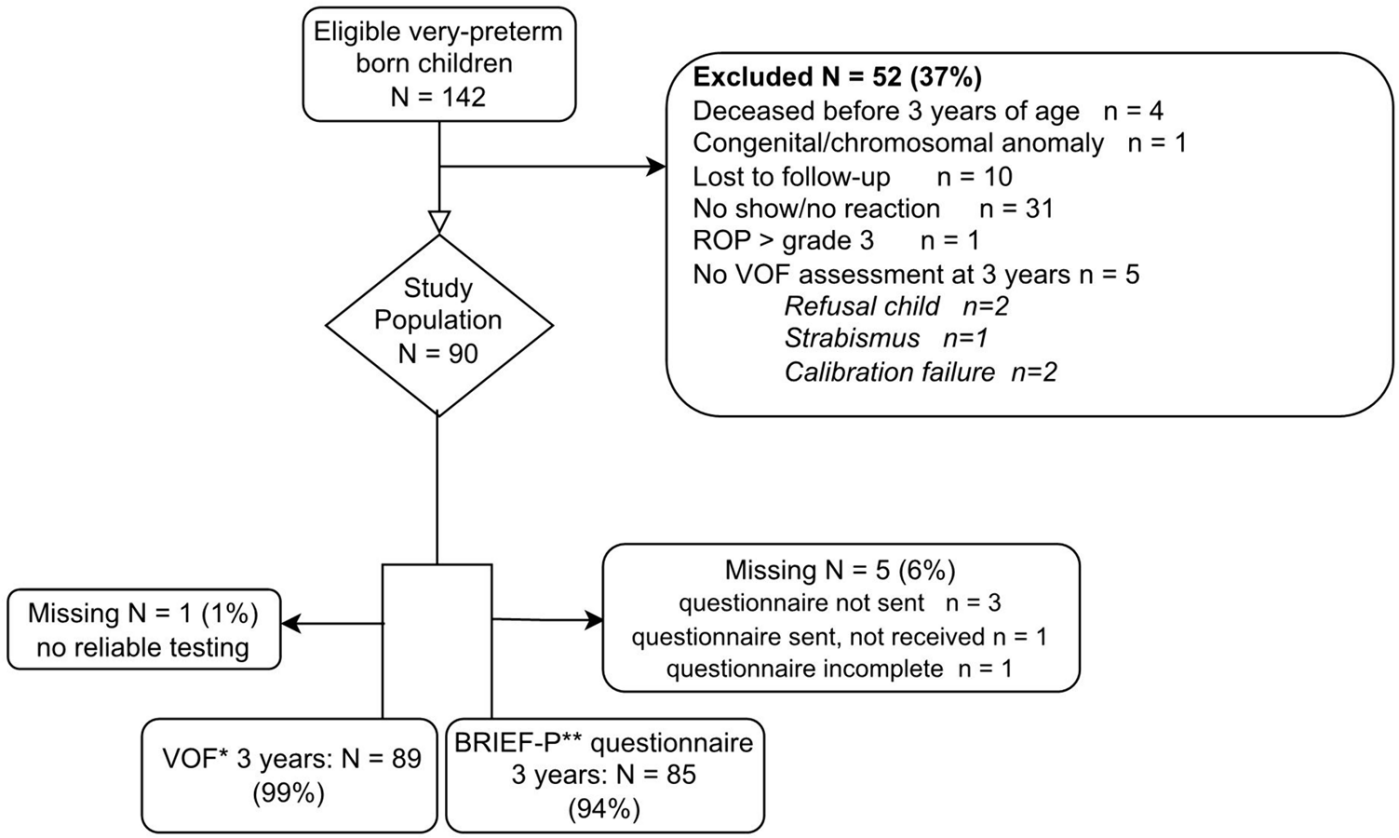
Flowchart of study population. *VOF visual orienting function, **BRIEF-P Behavior Rating Inventory of Executive Function, Preschool Version. *n* number, ROP retinopathy of prematurity

Ninety children were included (Fig. 1), of whom the characteristics are shown in Table 1.

**Table 1:** Patient characteristics

	Total group (*N* = 90)	Normal VOF group (*n* = 61)	Abnormal VOF group (*n* = 28)	*p* value
Demographic characteristics				
Gestational age (weeks)	27 + 6 [26 + 5; 29 + 0]	27 + 6 [27 + 0; 29 + 0]	27 + 3 [25 + 4; 28 + 5]	0.07
Birth weight (grams)	1038 [829; 1263]	1065 [843; 1293]	995 [792; 1240]	0.43
Birth weight SDS	0.13 [− 0.40; 0.70]	0.12 [− 0.46; 0.75]	0.28 [− 0.18; 0.58]	0.39
Sex girl	39 (43)	26 (43)	12 (43)	0.98
Apgar 5 min	8 [6;9]*	8 [6;9]*	8 [6;9]*	0.89
Family background				
Education level**				
*Low*	11 (12)	7 (12)	5 (18)	0.62
*Middle*	28 (31)	20 (33)	7 (25)	
*High*	50 (55)	34 (56)	16 (57)	
Ethnicity: *Western European*	72 (80)	46 (75)	25 (89)	0.13
Maternal smoking (yes)	7 (8)***	5 (8)***	2 (7)	0.29
Maternal age (in years)	31 [27;34]	31 [27;36]	29.5 [26.25;32]	0.16
Neonatal morbidity				
IVH				
*No IVH*	73 (81)	49 (80)	23 (82)	0.84
*IVH grade 1*	10 (11)	8 (13)	2 (7)	
*IVH grade 2*	7 (8)	4 (7)	3 (11)	
Cerebellar bleeding (yes)	2 (2)	0 (0)	2 (7)	**0.04**
PVL (yes)	2 (2)	0 (0)	2 (7)	**0.04**
BPD				
*No BPD*	56 (62)	40 (65)	15 (54)	0.28
*Mild BPD*	21 (23)	15 (25)	6 (21)	
*Severe BPD*	13 (14)	6 (10)	7 (25)	
ROP				
*No ROP*	50 (55)	36 (59)	13 (46)	0.27
*ROP grade 1*	28 (31)	16 (26)	12 (43)	
*ROP grade 2*	7 (8)	6 (10)	1 (4)	
*ROP grade 3*	5 (6)	3 (5)	2 (7)	
NEC				
*No NEC*	84 (94)	59 (96)	24 (85)	0.05
*NEC stage I*	1 (1)	0 (0)	1 (4)	
*NEC stage II*	2 (2)	1 (2)	1 (4)	
*NEC stage III*	3 (3)	1 (2)	2 (7)	
Characteristics at 3 years				
CP, of which:	3 (3)	1 (1)	2 (8)	0.17
GMFCS level 1	3 (3)	1 (1)	2 (8)	
Visual disorders, of which:	5 (6)	4 (7)	1 (4)	0.60
Wearing glasses	5 (6)	4 (7)	1 (4)	0.56
Strabismus	4 (5)	2 (3)	2 (8)	0.39
Nystagmus	0 (0)	0 (0)	0 (0)	NA

Data are presented for the total sample and for the subgroups with abnormal and normal VOF as median [interquartile range] or *n* (%); one patient was not categorized due to lack of reliable testing results. Abnormal VOF was defined as having one or more of the four stimuli being delayed (RTF above + 2 SD of the reference). *P* values for comparisons using Mann Whitney U or chi-square tests

*BPD* bronchopulmonary dysplasia, *CP* cerebral palsy, *GMFCS* Gross Motor Function Classification System, *n* number, *NA* not applicable, *NEC* necrotizing enterocolitis, *PVL* periventricular leukomalacia, *ROP* retinopathy of prematurity, *SDS* standard deviation score, *IVH* intraventricular hemorrhage

^a^Missing *n* = 1

^b^Missing *n* = 1

^c^Unknown *n* = 5

### VOF

Visual orienting data were available for 89 children (99%) and are presented in Table 2. The attention, fatigue, and mobility scores were all around 3, indicating scores slightly above average (“now and then”). For the cartoon and contrast stimuli, testing was reliable in 88% and 84% of the cases, with 19% and 6% of the children showing a delayed RTF on these stimuli, respectively. For Motion and Form, testing was reliable in 92% and 72%, with 5% and 9% showing a delayed RTF, respectively. Sixty-one children scored normal (below + 2 SD) at all stimuli, and the other 28 scored abnormal (+ 2 SD) at least one of the stimuli. Patient characteristics for both groups are shown in Table 1. None of the patient characteristics significantly differed between the groups, except for periventricular leukomalacia and cerebral bleeding being more common in the abnormal group (*p* = 0.04).

**Table 2:** Visual orienting function (VOF) parameters at 3 years

	3 years (*N* = 90)
Corrected age at measurement	3.21 [3.10; 3.55]
**Eye tracking feasibility factors**	
Attention score (1–5)	3.59 (0.77)
Fatigue score (1–5)	3.58 (0.90)
Restless/mobility score (1–5)	2.86 (0.92)
**Cartoon stimulus**^a^	
Number (%) of reliable tests*	79 (88%)
% of stimuli detected	65% [56; 80]
RTF (ms)	242 [230; 257]
Number delayed *n* (%)	15 (19%)
**Contrast stimulus**^a^	
Number (%) of reliable tests	76 (84%)
% of stimuli detected	65% [52; 79]
RTF (ms)	324 [288; 379]
Number delayed compared to term peers *n* (%)	6 (8%)
**Motion stimulus**^a^	
Number (%) of reliable tests	83 (92%)
% of stimuli detected	79% [62; 85]
RTF (ms)	493 [437; 612]
Number delayed compared to term peers n (%)	4 (5%)
**Form stimulus**^a^	
Number (%) of reliable tests	65 (72%)
% of stimuli detected	71% [54; 79]
RTF (ms)	651 [528; 804]
Number delayed compared to term peers *n* (%)	6 (9%)

A cartoon test was reliable if more than three stimuli were seen. Count values are shown as absolute numbers (percentage), feasibility factors are shown as mean (SD score), reaction times to fixation, and % of stimuli detected are shown as median [interquartile range]. Reaction times to fixation (RTF) and number of delayed were only calculated for reliable tests. Number and patterns of delay represent comparisons with the normative RTF references

*N* number, *ms* milliseconds, *RTF* reaction times to fixation, *SD* standard deviation, *VOF* visual orienting function

^a^Missing *n* = 1 (due to no reliable tests)

### EF

**Fig. 2:**
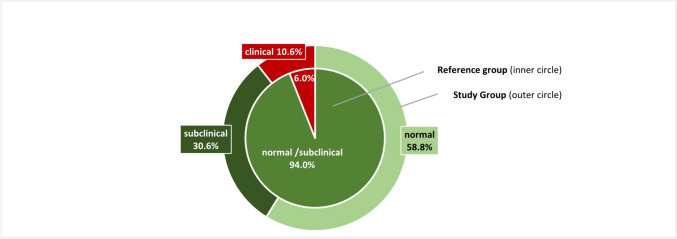
Global Executive Composite score. Classification of Global Executive Composite score for the Dutch Reference (inner circle) and for the study population (outer circle); classified as “normal” (*T*-score < 60), “subclinical” (60–65), and “clinical” (> 65)

Complete BRIEF-P questionnaire results were available in 85 (94%) of cases (Fig. 1). Table 3 shows that the mean Global Executive Function Composite score was 49.41 (SD 11.87). Fifty-nine percent of the parents reported a normal composite score, 31% a subclinical, and 10% a clinical (*T*-score ≥ 65) composite score (Table 3; Fig. 2). For the five subscales, the percentage of scores within the clinical range was 11% (shift and working memory), 12% (plan/organize), and 14% (inhibit and emotional control). Clinical scores for the three indexes ranged from 9% (inhibitory self-control) to 11% (emergent metacognition) and 18% (flexibility).

**Table 3:** Executive functioning (BRIEF-P, scaled scores) outcome of the study population at 3 years

	Study population (*N* = 85)
**BRIEF-P scores**^a^	
Inhibit	48.09 (11.41)
*% Clinical score*	14%
Shift	50.21 (12.64)
*% Clinical score*	11%
Emotional control	51.20 (11.84)
*% Clinical score*	14%
Working memory	49.62 (11.51)
*% Clinical score*	11%
Plan/organize	49.33 (11.15)
*% Clinical* score	12%
**BRIEF-P P Global Executive Composite score**	49.41 (11.87)
*% Clinical score*	11%
**BRIEF-P scales**	
Inhibitory self-control	49.15 (11.29)
*% Clinical score*	9%
Flexibility^b^	50.93 (12.89)
*% Clinical score*	18%
Emergent metacognition	49.36 (11.12)
*% Clinical score*	11%
BRIEF-P negativity score	0 [0;0]
BRIEF-P inconsistency score	3 [2;5]

Executive functioning (EF) scores are shown as mean (SD) and absolute numbers (percentage). Negativity score and inconsistency score are shown as median [interquartile range]. Negativity score ranges from 0 to 6 and inconsistency score ranges from 0 to 10. Clinical score is a T-score at or above 65

*BRIEF-P* Behavior Rating Inventory of Executive Function, Preschool Version

^a^Higher scores indicate more difficulties with EF

^b^Flexibility *n* = 81

### VOF and EF

Eighty-four children were included in the multiple regression analyses. Abnormal VOF was not associated with BRIEF-P Global Executive Composite score, nor with any of the three scale or five subscale executive function scores (Table 4). Adjusting for age at assessment, sex, birth weight, and parental education did not change the results.

**Table 4:** Visual orienting function and executive functioning

	*Normal VOF group (n* = *58)*	*Abnormal VOF group*^*a*^* (n* = *26)*	*Β*_*basic*_ *(95% CI)*	*P*_*basic*_	*Β*_*adjusted*_ *(95% CI)*	*P*_*adj*_
BRIEF-P inhibit score	46.0 [38.8; 52.0]	48.0 [40.8; 56.3]	2.65 (− 2.73; 8.03)	0.33	2.40 (− 2.97; 7.77)	0.38
*n (%) subclinical score*	8 (13)	4 (14)				
*n (%) clinical score*	7 (12)	5 (18)				
BRIEF-P shift score^b^	47.5 [39.8; 58.8]	47.0 [42.0; 58.5]	2.51 (− 3.60; 8.63)	0.42	2.38 (− 3.78; 8.55)	0.44
*n (%) subclinical score*	6 (10)	3 (11)				
*n (%) clinical score*	17 (28)	7 (25)				
BRIEF-P emotional control score	48.5 [39.0; 58.5]	50.0 [42.0; 53.0]	− 1.31 (− 6.97; 4.36)	0.65	− 1.41 (− 7.15; 4.32)	0.63
*n (%) subclinical score*	9 (15)	3 (11)				
*n (%) clinical score*	18 (30)	9 (32)				
BRIEF-P working memory score	49.0 [40.0; 54.0]	47.0 [42.5; 63.5]	3.75 (− 1.71; 9.22)	0.18	3.44 (− 1.97; 8.84)	0.21
*n (%) subclinical score*	4 (7)	5 (18)				
*n (%) clinical score*	22 (36)	6 (21)				
BRIEF-P plan/organize score^b^	47.0 [41.0; 53.8]	49.0 [42.0; 56.0]	1.05 (− 4.36; 6.46)	0.70	0.92 (− 4.54; 6.37)	0.74
*n (%) subclinical score*	8 (13)	2 (7)				
*n (%) clinical score*	10 (16)	7 (25)				
Global Executive Composite score	48.0 [40.0; 55.3]	47.0 [39.5; 59.0]	2.18 (− 3.45; 7.80)	0.09	1.91 (− 3.72; 7.53)	0.50
*n (%) subclinical score*	19 (33)	7 (27)				
*n (%) clinical score*	5 (9)	4 (15)				
Inhibitory self-control scale	46.0 [40.0; 57.0]	49.0 [40.0; 53.8]	1.23 (− 4.13; 6.59)	0.65	1.01 (− 4.36; 6.39)	0.71
*n (%) subclinical score*	4 (7)	4 (14)				
*n (%) clinical score*	19 (31)	6 (21)				
Flexibility scale^c^	48.0 [40.5; 58.0]	46.0 [43.0; 62.0]	0.49 (− 5.97; 6.95)	0.88	0.18 (− 6.39; 6.76)	0.96
*n (%) subclinical score*	8 (13)	7 (25)				
*n (%) clinical score*	20 (33)	4 (14)				
Emergent metacognition scale	49.0 [41.5; 53.5]	48.0 [40.8; 62.0]	2.66 (− 2.61; 7.94)	0.32	2.40 (− 2.86; 7.65)	0.37
*n (%) subclinical score*	5 (8)	4 (14)				
*n (%) clinical score*	15 (25)	4 (14)				

Executive functioning (EF) scores in the abnormal versus normal visual orienting function (VOF) group. Shown are group median [interquartile range], number (%), effect estimates, and 95% CI of the comparison between the normal (0) and abnormal (1) VOF group based on linear regression analysis adjusted for age at assessment (basic model). The confounder model was adjusted for age at assessment, sex, birth weight, and parental education level

^a^Abnormal is classified as 1 or more of the 4 stimuli being delayed as compared with the normative RTF references

^b^*n* = 25 in abnormal VOF group

^c^*n* = 23 in abnormal VOF group and 57 in normal group. EF is measured with the BRIEF-P questionnaire. Higher scores indicate more difficulties with EF. Average score is a score below 60. Subclinical score is a *T*-score between 60 and 65, Clinical score is a *T*-score 65 or above

*BRIEF-P* Behavior Rating Inventory of Executive Function, Preschool Version, *CI* confidence interval, *n* number, *VOF* visual orienting function

The predefined explorative analysis showed an association between abnormal VOF and two of the eight selected individual EF items. Children with abnormal VOF were at higher odds to score “often” at the item “easily sidetracked”; adjusted odds ratios (aORs) 4.18 ((CI: 1.21–14.41), *p* = 0.02), as well as at the item “short attention span”; aOR 4.52 ((CI: 1.34–15.22), *p* = 0.02) (Table 5).

**Table 5:** Visual orienting function and BRIEF-P item analysis

*Subscales and item*	*Normal VOF group (n* = *58)*	*Abnormal VOF group*^*a*^* (n* = *26)*	*Exp(B)*_*basic*_ *95% CI*	*P*_*basic*_	*Exp(B)*_*adjusted*_ *95% CI*	*P*_*adjusted*_
**Inhibit**						
Item 58: Easily sidetracked yes	8 (15)	9 (35)	3.65 (1.17; 11.32)	**0.03**	4.18 (1.21; 14.41)	**0.02**
**Shift**						
Item 25: Bothered by loud noises yes	9 (16)	2 (8)	0.50 (0.10; 2.52)	0.40	0.48 (0.09; 2.45)	0.37
**Working memory**						
Item 7: Trouble complete tasks yes	0 (0)	0 (0)	NA	NA	NA	NA
Item 12: Trouble concentrating yes	5 (9)	6 (23)	3.54 (0.94; 13.39)	0.06	4.01 (0.98; 16.44)	0.05
Item 61: Short attention span yes	7 (13)	9 (35)	4.45 (1.37; 14.50)	**0.01**	4.52 (1.34; 15.22)	**0.02**
**Plan/organize**						
Item 19: Cannot find things yes	1 (2)	2 (8)	9.13 (0.45; 185.9)	0.15	9.86 (0.45; 217.6)	0.15
Item 39: Caught up in small details yes	2 (3)	1 (4)	1.71 (0.12; 23.73)	0.69	1.69 (0.12; 24.23)	0.70
Item 44: Cannot find things yes	0 (0)	3 (12)	NA	NA	NA	NA

Exploratory analysis of selected BRIEF-P executive functioning items in the abnormal versus normal visual orienting function (VOF) group. Shown are number (%) and odds ratio’s (Exp(B)) with 95% confidence interval of the comparison between the normal VOF group (0) and abnormal group (1) based on logistic regression analysis adjusted for age at assessment (basic model). The confounder/adjusted model is adjusted for age at assessment, sex, birth weight, and parental education level

*CI* confidence interval, *n* number, *NA* not applicable, *VOF* visual orienting function

^a^Abnormal is classified as 1 or more of the 4 stimuli being delayed as compared with the normative RTF references

## Discussion

In our population of children born < 30 weeks of gestation, we observed a higher percentage of parent-reported EF problems compared to Dutch reference norms (10% vs. 6%) [28] and a moderate to high percentage (between 5% and 19%) of children with abnormal VOF at 3 years CA. While no direct associations were observed between VOF and overall EF scores, delays in VOF were linked to parent-reported problems that related to attention and concentration span.

### Visual orienting function

A relatively high prevalence of VOF delays in RTF was found at 3 years, especially for the cartoon stimuli (19%). VOF measures reflexive reactions to visual stimuli, showing decreasing reaction times during normal development [24], indicating better alertness and faster attention orienting. However, very preterm children often show delayed VOF development at 1 and 2 years, deviating from this developmental trajectory [3, 14, 15]. VOF starts developing early, in the first year of life, along with the maturation of specific cortical visual systems. Visual and attentional function in general serves as the basis for early developing sensory-motor and cognitive skills during early childhood [31]. It is suggested that visual deficits in preterm children may be related to the cortical dorsal stream and its connections to parietal, frontal, and hippocampal areas [31]. Therefore, VOF delays as observed in the present study in children born preterm, may reflect a cluster of deficits connected to these areas. The viewing reactions, i.e., VOF, could be a potential qualitative marker of visual information conduction, in the sense that better-developed cerebral connectivity could allow for faster viewing reactions but also for faster cognitive processing.

### Executive functioning

Our study observed a high incidence of clinical scores among very preterm children, suggesting clinically significant EF difficulties. This aligns with the scarce number of previous studies in preterm children born < 32 weeks of GA, at the age of 3.5–4 years[2], which reported clinical *T*-scores on the BRIEF-P between 6 and 20% [32, 33]. Despite average mean scores and scales in our study, rates of clinical *T*-scores varied between 9% and 18%, exceeding the Dutch norms of 3% and 9% [28]. Notably, 14% of the children showed abnormal *T*-scores in inhibit and/or emotional control, surpassing Dutch norms (clinical *T*-score inhibit 8%, emotional control 3%) [28]. This is in line with another study, suggesting that preterm children exhibit more difficulties with inhibitory control and reward waiting at preschool age [2]. Additionally, 18% of the preterm children exhibited abnormal “Flexibility” *T*-scores (shifting and emotional self-control scales), against 5% in the reference population [28], indicating challenges in behavioral and emotional regulation during unexpected events. This is consistent with findings that preterm born toddlers struggle with emotion regulation [2]. Overall, preterm born children are at higher risk of EF difficulties, already at preschool age. This is particularly relevant given that these early difficulties are associated with later behavior and learning problems, weaknesses in social cognition, and poorer academic achievement [2].

### Associations between visual orienting function and executive functioning

A previous study showed that the most vulnerable visual and visuomotor functions in preterm born children involve the allocation of attention and the selection and executive control of behavior [34]. Despite the lack of associations between abnormal VOF and overall EF scores, we found in our secondary analysis that children with abnormal VOF had a higher likelihood of focusing of attention and concentration problems. According to Posner and Petersen’s neurocognitive model [35], attention involves three neural networks: alerting, orienting, and executive control, and each network follows different developmental trajectories. Alerting and orienting develop in childhood and executive control through early adolescence. Deficits in alerting were found in infant and adolescent preterms, while deficits in executive attention were observed from early childhood onwards [35]. Our data suggests that already in early childhood, a connection between delays in orienting responses and specific EF items such as attention and concentration span exists. Impairments in cortical, dorsal stream, and attention networks may underlie these attention and visual problems in preterm children [16, 31]. For example, hyperactive behavior and inattention increased the risk for visual-motor deficits in children born preterm < 37 weeks GA aged 5.5 years [34]. The effective performance of the networks appears to be lower with lower GA at birth [16].

In the current study, children who had at least one abnormal RTF had four times higher odds for attention and concentration span problems indicated by parents in daily activities. The clinical impact of early VOF testing, especially in case of abnormal RTF values, may increase awareness of caregivers for attention and concentration problems and understanding of a child’s functioning. In turn, such testing allows for early referral for targeted intervention programs during school age. Open questions include (1) the extent to which early differences in orienting and alerting networks provide the developmental basis for later executive attention and/or function differences, and (2) what other intermediate factors could play a role, and (3) what mechanistic role plays processing speed and attention mechanisms in executive functioning in children born very preterm? A two-step approach as carried out in the present work provides ecologically valid and behavioral information that may be pivotal to interpreting later EF assessments [7].

### Strengths and limitations

Strengths of our study are the comprehensive approach to investigating links between VOF and EF in children born very preterm at the same time point, allowing for an in-depth analysis of the problem. The associations we found were based on both expert opinion and statistical testing, to reduce the likelihood of chance findings. With this strategic approach, we focused on clinically relevant items and minimized the potential for type I errors. Our study faced limitations due to the fact that the VOF paradigm is not designed to assess EF measures, which led to rely on item-level comparisons. Furthermore, we excluded children with ROP higher than grade 3, aligning with some [3, 14] but not all [15] previous studies. We assumed this had minimal impact on our findings given the small number of children with ROP grade 3 (*n* = 4) who had reliable VOF tests. Using questionnaires always creates a risk of recall or response bias. Since two BRIEF-P questionnaires were incomplete/not received, we expect this bias to be minimal. Assessment at a single time point limits understanding of long-term outcomes; therefore, longitudinal studies are recommended to explore the persistence of EF deficits and their impact on later academic achievement and daily functioning. Nevertheless, early identification of EF problems can favor awareness from a young age.

## Conclusions

Our study reveals that very preterm born children have a notable prevalence of abnormal VOF and exhibit more EF problems compared to Dutch reference norms. Although there was no direct correlation between VOF and overall EF scores, we found that children with abnormal RTF seem to face an increased risk for parent-reported attention and concentration difficulties. Early detection through eye-tracking tests can increase awareness and facilitate communication. Further research is needed to validate these findings and explore their long-term implications.

## Supplementary Information

Below is the link to the electronic supplementary material.

Supplementary file1 (DOCX 1632 KB)

## Data Availability

The data that support the findings of this study are available on request from the corresponding author, KFMJ.

## Abbreviations

BRIEF-P: Behavior Rating Inventory of Executive Function—Preschool Version
CA: Corrected age
CI: Confidence interval
EF: Executive functioning
GA: Gestational age
IVH: Intraventricular hemorrhage
ms: Milliseconds
NICU: Neonatal Intensive Care Unit
PHVD: Post-hemorrhagic ventricular dilatation
ROP: Retinopathy of prematurity
RTF: Reaction time to fixation
SDS: Standard deviation score
SD: Standard deviation
SGA: Small for gestational age
VOF: Visual orienting function
