# Supplementary material for: Visual attention and processing function in relation to executive functioning in very preterm–born children aged 3 years: a prospective cohort study
Source: Eur J Pediatr. 2024 Aug 17;183(10):4519–29. doi: 10.1007/s00431-024-05720-2 (PMC11413138; doi:10.1007/s00431-024-05720-2)
Supplement: Supplementary file 1 — Supplementary file1 (DOCX 1632 KB) [file 431_2024_5720_MOESM1_ESM.docx]

Supplementary Files


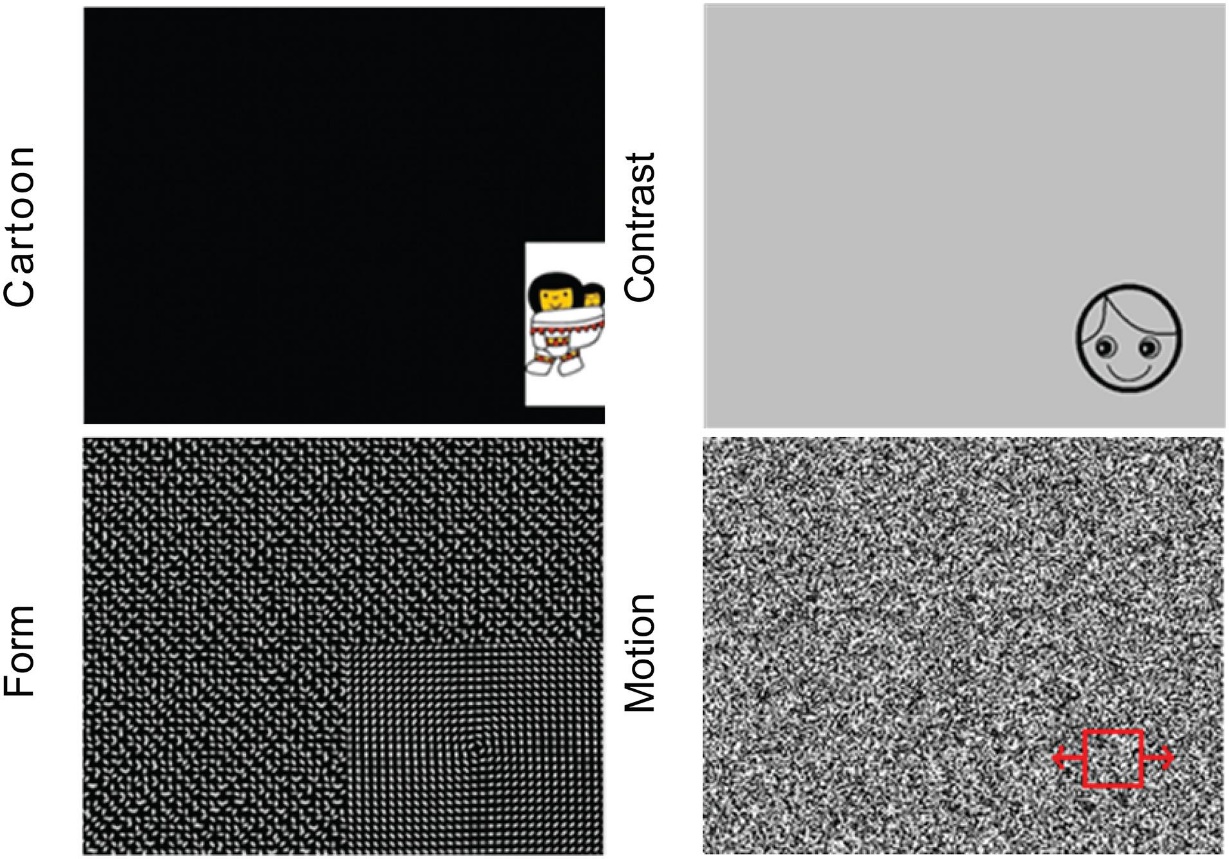


# Supplemental Figure S1: The four stimulus types with various visual content as presented with the eye tracking-based paradigm (adapted from Kooiker et al. 2016 [21]).

# Supplemental Table S1: BRIEF-P Item Analysis

| **Item** | **N** | **Subscale** | **Item description** |
| --- | --- | --- | --- |
| 12 | 6 | Working Memory | Trouble concentrating |
| 44 | 6 | Plan/Organize | Cannot find things |
| 19 | 5 | Plan/Organize | Cannot find things |
| 61 | 5 | Working Memory | Short attention span |
| 7 | 4 | Working Memory | Trouble complete tasks |
| 25 | 4 | Shift | Bothered by loud noises |
| 39 | 4 | Plan/Organize | Caught up in small details |
| 58 | 4 | Inhibit | Easily sidetracked |

The item number, number of experts that rated this item as important, and item description are listed in this table. Items were mentioned by > 66% of the panel members. Abbreviations: N = number of experts mentioned this item.


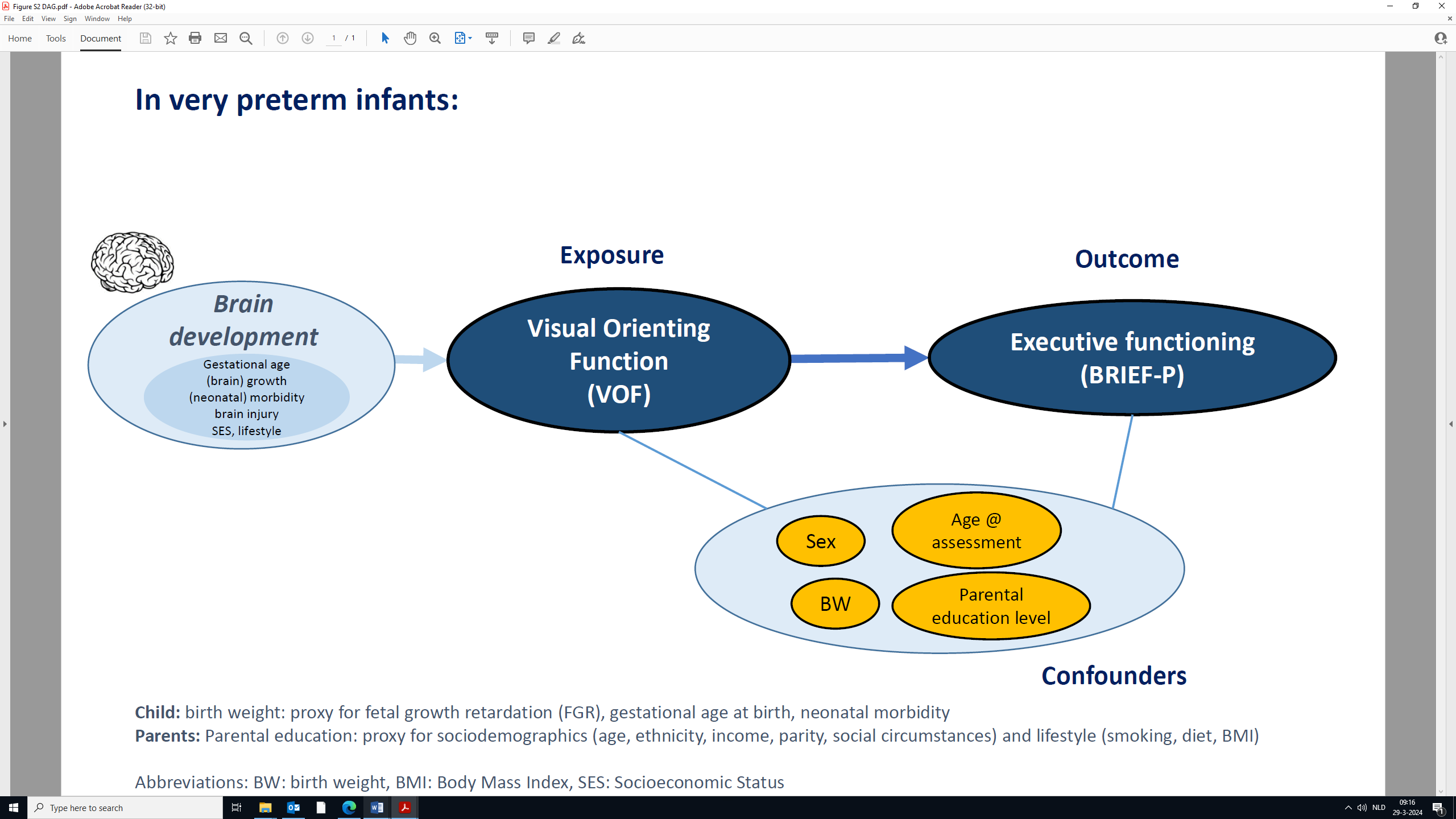


# Supplemental Figure S2: Directed Acyclic Graph (DAG)

This figure shows the DAG of the confounders included in our confounder model for regression analyses.
